# Statins Have an Anti-Inflammation in CKD Patients: A Meta-Analysis of Randomized Trials

**DOI:** 10.1155/2022/4842699

**Published:** 2022-10-22

**Authors:** Jianting Wang, Ziwei Chen, Yuliang Qiu, Ling Wu, Huan Wang, Lisheng Wu, Liangbin Zhao, Dengpiao Xie

**Affiliations:** ^1^The Affiliated People's Hospital of Fujian University of Traditional Chinese Medicine, Fuzhou, Fujian province, China; ^2^Chengdu First People's Hospital, Chengdu, Sichuan Province, China; ^3^Hospital of Chengdu University of Traditional Chinese Medicine, Chengdu, Sichuan Province, China; ^4^Southern Medical University, Guangzhou, Guangdong Province, China

## Abstract

**Background:**

Persistent inflammation has been recognized as an important comorbid condition in patients with chronic kidney disease (CKD) and is associated with many complications, mortality, and progression of CKD. Previous studies have not drawn a clear conclusion about the anti-inflammatory effects of statins in CKD. This meta-analysis is aimed at assessing the anti-inflammatory effects of statins therapy in patients with CKD.

**Methods:**

A comprehensive literature search was conducted in these databases (Medline, Embase, Cochrane library, and clinical trials) to identify the randomized controlled trials that assess the anti-inflammatory effects of statins. Subgroup, sensitivity, and trim-and-fill analysis were conducted to determine the robustness of pooled results of the primary outcome.

**Results:**

25 eligible studies with 7921 participants were included in this meta-analysis. The present study showed that statins therapy was associated with a decreased C-reactive protein (CRP) (-2.06 mg/L; 95% CI: -2.85 to -1.27, *p* < 0.01). Subgroup, sensitivity, and trim-and-fill analysis showed that the pooled results of CPR were stable.

**Conclusion:**

This meta-analysis demonstrates that statins supplementation has anti-inflammatory effects in patients with CKD. Statins exert an anti-inflammatory effect that is clinically important in improving complications, reducing mortality, and slowing progression in CKD. We believe that the benefits of statins to CKD are partly due to their anti-inflammatory effects. However, stains usually are prescribed in the CKD patients with dyslipidemia, whether statins can reduce inflammation in CKD patients with normal serum lipid needed to explore in the future. Therefore, we suggest that randomized clinical trials need to assess the effect of statins in CKD patients with normal serum lipid. Whether statins can be prescribed for aiming to inhibit inflammation in CKD also needed further study. *Trial Registration*. The study protocol was registered in the International Prospective Register of Systematic Reviews (PROSPERO); registration number: CRD42022310334.

## 1. Introduction

Chronic kidney disease (CKD) has been recognized as an important public problem in the world. The prevalence of CKD is about 13.4% worldwide [1], and more than 1 in 7, which is 15% of American adults, are estimated to have CKD based on the centers for disease control and prevention in 2021. The number of patients with CKD is estimated to be 120 million in China [2]. CKD was demonstrated as a major risk factor for all-cause mortality, and mild elevation of serum creatinine was associated with the increased death from any cause [3–5]. Persistent inflammation has been recognized as a common and important comorbid condition in patients with CKD, particularly in dialysis patients, which are responsible for cardiovascular and all-cause mortality [6]. The progression of CKD is closely associated with systemic inflammation [7, 8]. In addition, clinical researches have proved that elevated inflammatory markers, C-reactive protein (CRP), and interleukin-6 (IL-6) were associated with many complications of CKD, such as malnutrition, atherosclerosis, insulin and erythropoietin resistance, coronary artery calcification, heart disease, mineral and bone disease, anemia, and enhanced CKD mortality [6, 9–12]. Therefore, inhibiting inflammation in CKD would bring many benefits to these patients. The management of chronic inflammation in CKD includes improvement of malnutrition, correction of anemia and vitamin D deficiency, adequate dialysis, and exercise [6, 13]. However, there is no clear evidence showing these approaches can significantly improve inflammation state in CKD. Therefore, it is necessary to find an effective therapy for the management of inflammation in CKD.

CKD is strongly associated with dyslipidemia, characterized by elevated low-density lipoprotein (LDL) cholesterol and triglycerides, and decreased high-density lipoprotein (HDL) cholesterol [14]. Patients with CKD have dyslipidemia even at the early stages of CKD, and dyslipidemia often worsens with the progression of CKD. The prevalence of dyslipidemia was 45.5% in CKD stage 1 and increased to 67.8% in CKD stage 4 [15]. Several mechanisms might explain the high prevalence of dyslipidemia in CKD. Hypertriglyceridemia is associated with a decreased renal function, which results in impairing clearance of triglyceride-rich lipoproteins [16]. In addition, decreased activity of lipoprotein lipase and hepatic triglyceride lipase are observed in CKD, leading to decreased fractional catabolic rate of triglycerides [17]. Elevated LDL might due to alteration of LDL receptor and reduced affinity of LDL to its receptor in CKD [18]. Low HDL is partly due to the reduction biosynthesis of apo-AI in the uremic milieu, since apo-AI is primary component of HDL [19]. Other studies showed that lecithin-cholesterol acyltransferase (LCAT) is important in HDL-mediated cholesterol uptake from extrahepatic tissues and influences levels and maturation of HDL. The levels and activity of LCAT decreased as the progression of CKD, which also account for diminished HDL [16, 20].

Statins, HMG-CoA reductase inhibitors, are a class of lipid-lowing drugs [21] that are the mainstay treatment for hyperlipidemia [22]. Statins have been widely used in CKD, and studies found that stains were used in 35.7% of CKD patients in USA [23] and 62.1% in veterans with CKD [24]. Studies have demonstrated that statins are effective in improving dyslipidemia in CKD [25]. The use of statins also are associated with attenuating the progression of renal function, declined proteinuria, and reduced cardiovascular mortality in CKD [26, 27]. Therefore, statins are one of important parts in management of CKD. Statins are also demonstrated to have another important pharmacological effect and anti-inflammation. Statins have been identified as potential drugs for attenuating inflammation in CKD. Statins exert an anti-inflammatory effect that might be related to lower cholesterol because cholesterol strongly promotes inflammation [28]. In addition, statins are associated with reduced activation of immune cells, such as T cells and monocyte [29]. In vivo studies, the results showed that statins improved lung injury and atherosclerosis due to their anti-inflammatory actions [30, 31]. Clinical studies showed that statins reduced inflammatory markers in patients with cardiovascular diseases, which are associated with reduced cardiovascular events [32, 33]. In addition, statins were demonstrated to ameliorate inflammation in CKD rats. Clinical studies also observed an association between statins and anti-inflammatory effects in CKD [34, 35]. Although some studies have assessed the anti-inflammatory effects of statins in patients with CKD; however, there is lacking conclusive evidence that statins have an anti-inflammatory effects in patients with CKD. Meta-analysis of randomized clinical trials combines different studies into one large study to increase statistical power and precise estimate of the effect size, which can draw a clear evidence. As far as we know, there is no meta-analysis focusing on the assessment of the anti-inflammatory effects of statins in patients with CKD. Therefore, we aimed at performing a meta-analysis of randomized clinical trials to assess the anti-inflammatory of statins in patients with CKD.

## 2. Methods

The present review was conducted and reported by Preferred Reporting Item for Systematic Reviews and Meta-analysis (PRISMA) [36]. The study protocol was registered in the International Prospective Register of Systematic Reviews (PROSPERO); registration number: CRD42022310334.

### 2.1. Data Sources and Search Strategies

Clinical trials were searched in the following databases: Embase, Medline, Cochrane Central Register of Controlled Trials, and Clinical Trial Registries with the search deadline of January 2022. The following keywords were used: “HMG-CoA reductase inhibitors”, “statin”, “fluvastatin”, “rosuvastatin”, “atorvastatin”, “lovastatin”,“simvastatin”, “pravastatin”, “cerivastatin” “inflammation”, “C-reactive protein”, “interleukin”, “tumor necrosis factor”, “chronic kidney failure”, “chronic renal insufficiency”, “chronic renal disease”, and “random^∗^ controlled trial”. Language is limited to English. In addition, to search the potential relevant trials, the references of similar clinical studies or reviews were reviewed.

### 2.2. Study Selection, Data Extraction, and Quality Assessment

**Figure 1:**
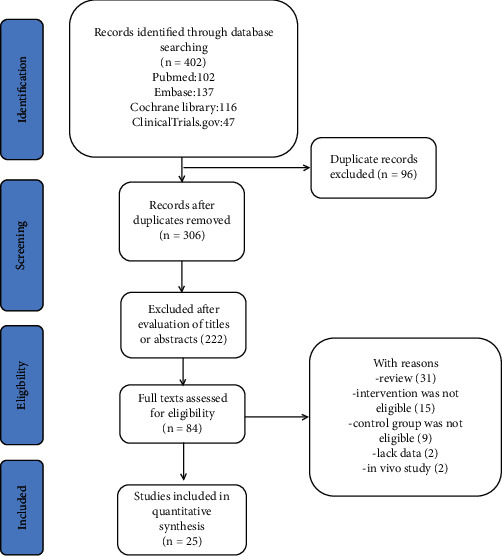
Flow of the search strategy and included studies.

Two independent reviewers assessed the titles and abstracts, and screened the full-text versions of the relevant trials. Disagreements were resolved by consensus between the reviewers, and if necessary, consulting with other reviewers. The studies were considered to be eligible if they assessed the effects of statins compared with placebo or conventional therapy in CKD, and the studies are randomized trials that reported inflammatory markers, such as CPR, hs-CRP, IL-6, and TNF-*α*. Patients receiving kidney transplantation were excluded. Reviews, case reports, letters, abstracts, and ongoing clinical trials without results were excluded. The flow diagram of study selection was outlined in Figure 1.

Each eligible trial was extracted into a spreadsheet. Two reviewers independently extracted the patient characteristics, including the stage of CKD, type of statins, the dose of the drug, follow-up duration, values of inflammatory markers at baseline and the end of treatment, and adverse events. Study quality was assessed by the Cochrane Risk of Bias Tool, which contains selection bias, performance and detection bias, attrition bias, reporting bias, and other sources of bias, and each component was identified as having a low, high, or unclear risk of bias. The third author resolves the discrepancies. The corresponding author is responsible for obtaining missing information and unpublished data.

### 2.3. Outcome Definition

Primary outcomes were defined as the change of CRP or hs-CRP from baseline to end of treatment. Secondary outcomes include the change of IL-6 and TNF-*α* from baseline to end of treatment.

### 2.4. Data Synthesis and Analysis

The effect size was assessed by weighted mean differences (WMDs) for continuous outcomes with a 95% confidence interval (CI). If clinical outcomes were measured more than once in the study, the data with the longest follow-up period was included. In addition, if the trial compared multiple treatment arms with the control group, the number of patients in the control group was divided by the number of the treatment arms. If data were reported as median, interquartile range, 95% CI, or standard error (SE), the data were converted to mean and standard deviation (SD) by the formula [37, 38]. If results were not significantly heterogeneous, a fixed-effect model was used, otherwise, a random-effect model was used. The heterogeneity of pooled results was assessed with *I*^2^ statistics. The value of *I*^2^ is from 0 to 100% (*I*^2^ > 50%, substantial heterogeneity; *I*^2^ = 25 − 50%, moderate heterogeneity; *I*^2^ < 25%, low heterogeneity). The possibility of publication bias for the primary outcome was assessed by the regression test of the Egger test and by a visual estimate of the funnel plot. Sensitivity analysis was conducted by omitting one study at a time and removing the studies with participants less than 40. The trim-and-fill method was conducted to detect and adjust for publication bias. The data were assessed by Review Manager, version 5.3 (Oxford, UK) or Stata 15.

### 2.5. Additional Analysis

Subgroup analysis were performed based on standard CRP or hs-CRP test, the duration of the intervention (≤6 or >6 months), predialysis CKD patients or dialysis patients.

## 3. Results

### 3.1. Literature Selection and Study Characteristic

A total of 402 relevant studies were identified by the initial search, and 96 duplicate studies were removed. The rest of the 306 studies were carefully assessed by the titles and abstracts, and 222 studies were removed because they did not meet inclusion criteria. Finally, 84 full-text studies were further assessed in detail, and 59 articles were excluded for the following reason: 31 studies were excluded because they were reviews or meta-analysis, 15 studies were excluded because the treatment was not eligible, 9 studies were excluded because the control group was not eligible, 2 studies were excluded because they did not have target outcomes, and 2 studies were excluded because they were in vivo studies. Finally, 25 studies meet the inclusion criteria [39–63] (Figure 1).

The summary characteristics of studies included in this review were presented in Table 1. 25 studies with 7921 participants were included in this review. Sample sizes range from 13 to 3267 patients. Dialysis patients were included in 14 studies [39–42, 45, 46, 48, 51–55, 61, 63], and CKD patients were included in 11 studies [43, 44, 47, 49, 50, 56–60, 62]. Patients received fluvastatin in 3 studies [39, 43, 53], rosuvastatin in 7 studies [40, 45, 48, 51, 59, 60, 62], atorvastatin in 8 studies [44, 46, 47, 50, 52, 54, 56, 63], simvastatin in 4 studies [41, 55, 58, 61], lovastatin in 1 study [50], and pravastatin in 2 studies [42, 57]. The dosage of statins was ranged from 5 mg to 80 mg daily. The duration of treatment range was from 8 weeks to 48 months. 1 study has two arms of treatment [55]. Intention-to-treat analysis was used in 5 studies [45, 48, 51, 54, 59].

### 3.2. Risk of Bias Assessment

**Figure 2:**
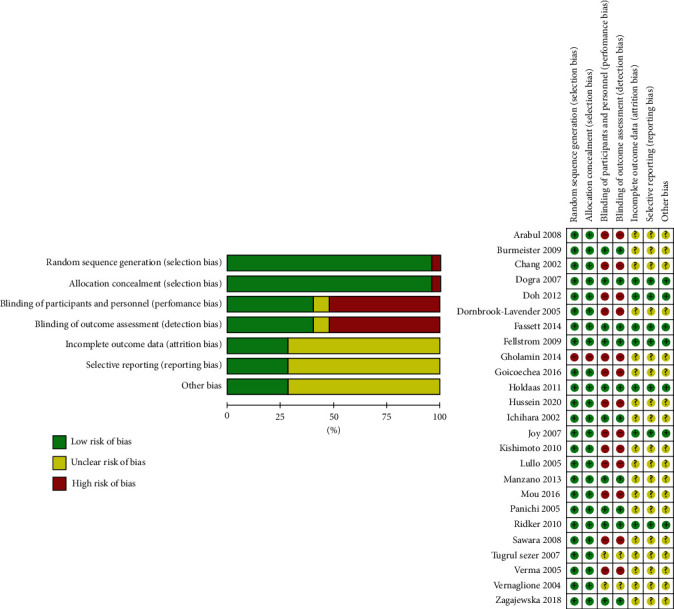
Summary of risk of bias.

Among the studies, 24 studies were considered as having a low risk of selection bias and 1 study was considered as having a high risk of selection bias. 10 studies were considered to have a low risk of performance and detection bias, 13 studies were considered to have a high risk of performance and detection bias, and 2 studies were considered to have an unclear risk of performance and detection bias. 7 studies were considered to have a low risk of attrition, reporting, and other bias. 18 studies were considered to have an unclear risk of attrition, reporting, and other bias. The details of assessments of the risk of bias were present in Figure 2.

### 3.3. Primary Outcome: CRP

**Figure 3:**
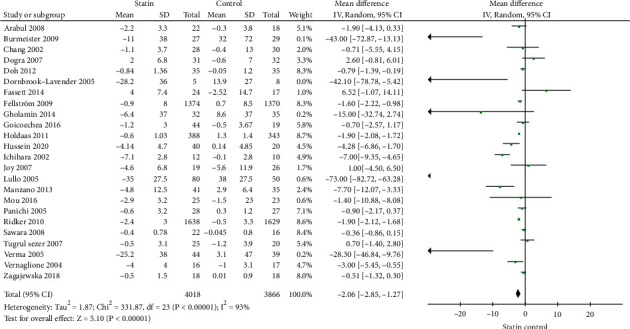
Forest plot for CRP.

A total of 24 studies [45–54, 56–63] were included in the meta-analysis for the CRP between the two groups. In the pooled analysis, the use of statins was associated with a significantly decreased CRP in patients with CKD (-2.06 mg/L; 95% CI: -2.85 to -1.27, *p* < 0.01, Figure 3).

### 3.4. Secondary Outcome: IL-6 and TNF-*α*

For IL-6, 8 studies [44, 45, 47, 50, 52, 55, 56, 61] were included in the meta-analysis, and the pooled results showed that there was no significant difference between the statin and control groups (0.1 pg/mL; 95% CI: -0.93 to 1.13, *p* = 0.85). For TNF-*α*, 4 studies [47, 50, 55, 61] were included in the meta-analysis, and the pooled results showed that there was no significant difference between the statin and control groups (-7.06 pg/mL; 95% CI: -14.49 to 0.38, *p* = 0.06).

### 3.5. Additional Analysis

**Figure 4:**
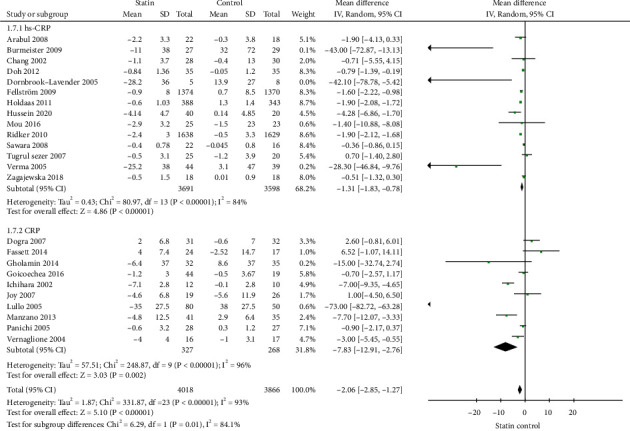
Subgroup analysis for CRP based on standard or high-sensitivity CRP test.

**Figure 5:**
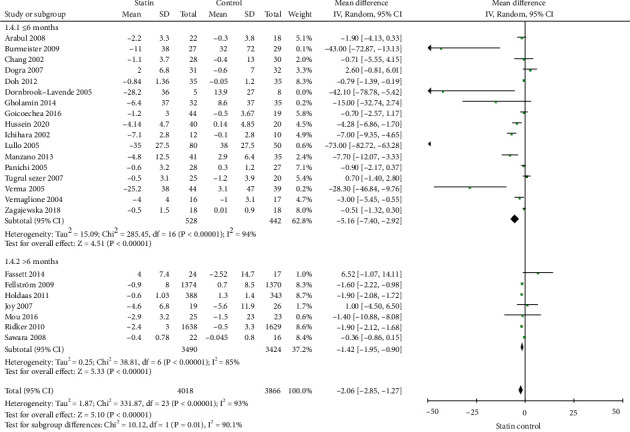
Subgroup analysis for CRP based on duration of statins therapy.

**Figure 6:**
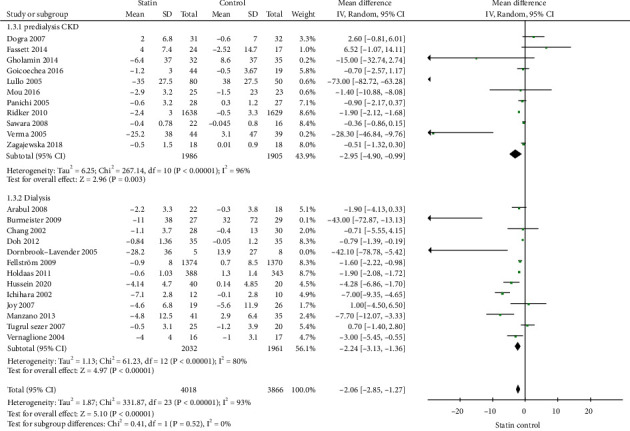
Subgroup analysis for CRP based on predialysis CKD or dialysis patients.

Subgroup analysis was performed based on the standard CRP or hs-CPR tests, study duration (≤6 months or >6 months), predialysis CKD, or dialysis patients. Statins use was associated with a significant decreased on levels of hs-CRP (-1.31 mg/L; 95% CI: -1.83 to -0.87, *p* < 0.01, Figure 4) and CRP (-7.83 mg/L; 95% CI: -12.91 to -2.76, *p* < 0.01, Figure 4). When the studies were stratified based on the study duration, statins use was associated with a significant decreased on CRP in the subgroup of studies with ≤6 months (-5.16 mg/L; 95% CI: -7.40 to -2.92, *p* < 0.01, Figure 5) or >6 months (-1.42 mg/L; 95% CI: -1.95 to -0.90, *p* < 0.01, Figure 5). When the studies were stratified into predialysis CKD patients or dialysis patients, statins use was associated with a significant decreased on CRP in the subgroup of studies with predialysis CKD patients (-2.95 mg/L; 95% CI: -4.90 to -0.99, *p* < 0.01, Figure 6) or with dialysis patients (-2.24 mg/L; 95% CI: -3.13 to -1.36, *p* < 0.01, Figure 6).

### 3.6. Sensitivity Analysis and Publication Bias

**Figure 7:**
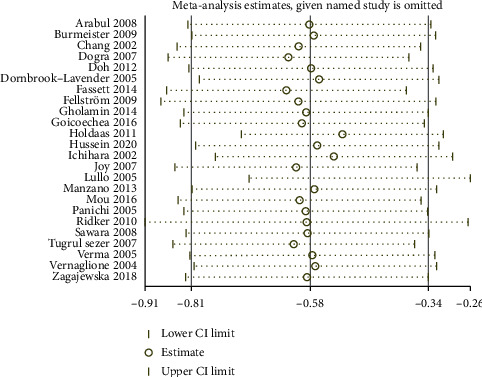
Leave-one-out forest plot for the CRP.

Sensitivity analysis was conducted including the leave-one-out method and removing studies with participants less than 40. Leave-one-out analysis showed that the pooled result was no significant change on CRP (Figure 7). Studies with participants less than 40 [46, 53, 56, 60, 63] were removed, and the pooled result was no significant change on CRP (-2.00 mg/L; 95% CI: -2.91 to -1.09, *p* < 0.01).

**Figure 8:**
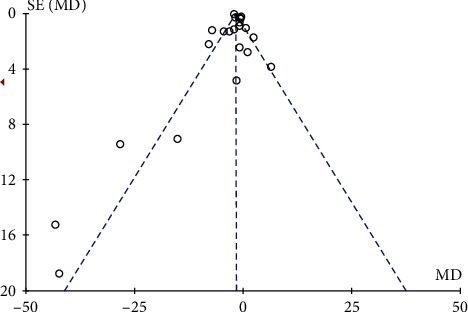
Funnel plot for CRP.

**Figure 9:**
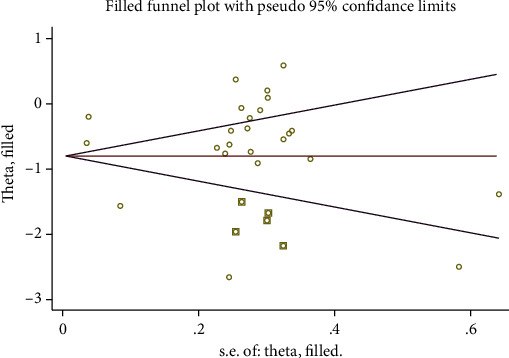
Trim-and-fill analysis for CRP.

Visual inspection of the funnel plots and Egger's tests were used to explore the potential publication. Egger's tests (*p* = 0.592) did not detect significant publication bias in the present meta-analysis of CRP. However, the funnel plot analysis showed some asymmetry by visual inspection (Figure 8). Therefore, the trim-and-fill method was conducted to detect and adjust for publication bias, and the adjusted result was no significant change on CRP (fixed model, -0.79 mg/L; 95% CI: -1.02 to -0.56, *p* < 0.01) or (random model, -0.56 mg/L; 95% CI: -0.6 to -0.51, *p* < 0.01) (Figure 9).

### 3.7. Adverse Events

All statins were well-tolerated. These studies reported adverse events [39, 40, 43, 44, 48, 51, 54, 57, 60]. There was no significant difference in any serious adverse events between treatment and control groups [39, 40, 43, 44, 48, 51, 54, 57, 60]. There was no significant difference in aminotransferase and creatinine concentration between treatment and control groups [48, 51, 59].

## 4. Discussion

The present meta-analysis aimed to assess the anti-inflammatory effects of statins in patients with CKD. To the best of our knowledge, this meta-analysis is the first one to explore this topic. The present meta-analysis data included 25 eligible randomized clinical trials with 7921 participants. Our meta-analysis showed that statins supplementation was significantly associated with a decreased CPR levels and did not significantly increase adverse events.

Chronic systemic inflammation is common in patients with CKD even in the early stage of CKD and is characterized by persistent, low to moderate levels of the increased inflammatory markers [64]. Many factors contribute to the chronic inflammatory state in CKD, including the increased production of pro-inflammatory cytokines, chronic and recurrent infection, oxidative stress and acidosis, alerted adipose tissue metabolism, and intestinal dysbiosis [12, 64]. Chronic inflammation is strongly associated with complications and progression of CKD and contributes to irreversible tubular injury and kidney failure [65]. CRP is the most common and important marker to reflect the inflammatory state in CKD. Compared with the control who did not have CKD, the serum level of CRP was significantly higher in patients with CKD. Studies have shown a positive correlation between CRP and serum creatinine in patients with CKD [66]. CRP has also been implicated in facilitating LDL deposition on the atrial wall and promotes atherosclerotic disease progression [67]. The level of CRP is associated with carotid intima-media thickness and endothelial function in patients with CKD [68, 69]. CRP has been demonstrated to be an independent risk factor of cardiovascular events and improved mortality prediction in CKD [70]. Therefore, considering the importance of CRP in CKD, CRP was used as the primary outcome in this meta-analysis.

Statins are prescribed extensively for cholesterol reduction in the protection against cardiovascular disease. Statins are also widely used and showed many beneficial effects in CKD. A retrospective study conducted by Sui et al. showed statin therapy improved the response of erythropoiesis-stimulating agents [71]. In addition, a previous meta-analysis showed that statin therapy reduces cardiovascular events in patients with CKD [72]. Another meta-analysis showed the beneficial effects of statin therapy in reducing microalbuminuria, proteinuria, and clinical deaths in CKD [73]. Recent convincing evidence suggests that the beneficial effects of statins apart from cholesterol reduction lie in their pleiotropic effects [74, 75]. Increasing evidence showed that statins have potential anti-inflammatory properties contributing to their beneficial effects in patients. The anti-inflammatory effects of statins have been proved to benefit patients with CKD or cardiovascular disease from clinical and laboratory studies [76, 77]. Our meta-analysis showed that statins therapy inhibited inflammatory state evidenced by reducing CRP levels which might be one of the mechanisms that contribute to beneficial effects in CKD. This finding provided clear evidence that statins therapy inhibited inflammation and may have important clinical significance in the management of CKD. Consistent with our results, a meta-analysis also demonstrated that statins inhibited inflammation in patients with metabolic syndrome [78]. Subgroup analysis showed that both predialysis CKD and dialysis patients have a significant reduction in CRP levels when those patients were treated with short- or long-term statins. However, we did not conduct additional subgroup analysis based on dose or types of statins due to the limited number of included studies. Therefore, more clinical studies are needed to explore the potential factors affecting their effects.

Underlying mechanisms of statins in anti-inflammatory effects are complicated in CKD and might be associated with the following reasons. First, accumulation evidence suggests NF-*κ*B activation is involved in the pathogenesis of persistent inflammation in CKD [79]. Activation of NF-*κ*B promotes the production of proinflammatory cytokines in CKD. Statins have been shown to inhibit the accumulation of NF-*κ*B in nuclear and further block the activation of its downstream targets, including proinflammatory cytokines [80, 81]. Second statins can inhibit NLRP3 inflammasome activation and IL-*β* maturation in mononuclear cells [82]. Third, statins can hamper macrophage differentiation into M1 subset and promote macrophage shift toward M2 subset [83], since M1 macrophages are mainly implicated in proinflammatory responses, while M2 macrophages are mainly implicated in anti-inflammatory responses [84]. Fourth, reactive oxygen species (ROS) play an important role in the inflammation in CKD [85]. Statins can inhibit NADPH oxidase activity by inhibiting Rac isoprenylation, increase NO production, and upregulate the expression and activity of ROS-scavenging enzymes, including catalase and superoxide dismutase, and these effects can reduce the ROS levels [83, 86]. Fifth, statins also have been shown to have an impact on immune cells. These studies showed that statins can inhibit activation and proliferation of T cells and macrophages and consequently suppress the inflammatory response [76, 87, 88].

Our meta-analysis had several important advantages. Some studies have been conducted to assess the relationship between statins supplementation and anti-inflammation in patients with CKD; however, the trials with sample size are small, the results are inconsistent, and conclusions are not convincing. The present meta-analysis review included 25 randomized studies with large samples of patients, which enhanced the statistical power to provide convincing results. The results of CRP are stable in sensitivity analysis, subgroups analysis, or trim-and-fill analysis. There were several limitations in our meta-analysis. First, we observed substantial heterogeneity in CRP using *I*^2^ statistics. We further conducted subgroups analysis to reduce heterogeneity based on treatment duration, predialysis CKD or dialysis patients, standard CRP or hs-CRP test; however, heterogeneity did not significantly reduce. Second, we found no significant publication bias in Egger's test in CRP; however, funnel plot analysis showed some asymmetry. Therefore, publication bias might exist. Third, the dose and types of statins used in included trials varied, and we did not further conduct subgroup analysis based on the dose and types of statins.

## 5. Conclusion

In conclusion, this meta-analysis demonstrates that statins supplementation has anti-inflammatory effects in patients with CKD. Statins exert an anti-inflammatory effect that is clinically important in improving complications, reducing mortality, and slowing progression in CKD. We believe that the benefits of statins to CKD are partly due to their anti-inflammatory effects. However, stains usually are prescribed in the CKD patients with dyslipidemia, whether statins can reduce inflammation in CKD patients with normal serum lipid needed to explore in the future. Therefore, we suggest that randomized clinical trials need to assess the effect of statins in CKD patients with normal serum lipid. Whether statins can be prescribed for aiming to inhibit inflammation in CKD also needed further study.

## Figures and Tables

**Table 1:** Basic characteristics of subjects and treatments of trials.

Reference	No. of patients (statins/control)	Type of patient	Interventions		Duration
			Statins	Control	
Arabul et al. [39]	40 (22/18)	Hemodialysis or peritoneal dialysis	Fluvastatin (40 mg twice daily)	Placebo	8 weeks
Burmeister et al. [40]	56 (27/29)	Hemodialysis	Rosuvastatin (10 mg daily)	Placebo	3 months
Chang et al. [41]	58 (28/30)	Hemodialysis	Simvastatin (20 mg daily)	Control	8 weeks
Dogra et al. [44]	63 (31/32)	CKD (3-5)	Atorvastatin (40 mg daily)	Placebo	6 weeks
Doh et al. [45]	70 (35/35)	Peritoneal dialysis	Rosuvastatin (10 mg daily)	Control	6 months
Dornbrook-Lavender et al. [46]	13 (5/8)	Hemodialysis	Atorvastatin (10 mg daily)	Control	20 weeks
Fassett et al. [47]	41 (24/17)	Serum creatinine > 120 *μ*mol/L	Atorvastatin (10 mg daily)	Placebo	3 years
Fellström et al. [48]	2744 (1374/1370)	Hemodialysis	Rosuvastatin (10 mg daily)	Placebo	1 year
Gholamin et al. [49]	67 (32/35)	CKD 3	Lovastatin (20 mg daily)	Placebo	3 months
Goicoechea et al. [50]	63 (44/19)	CKD (2-4)	Atorvastatin (20 mg daily)	Control	6 months
Holdaas et al. [51]	731 (388/343)	Hemodialysis	Rosuvastatin (10 mg daily)	Placebo	1 year
Hussein et al. [52]	60 (40/20)	Hemodialysis	Atorvastatin (20 mg daily)	Control	6 months
Ichihara et al. [53]	30 (15/15)	Hemodialysis	Fluvastatin (20 mg daily)	Placebo	6 months
Kishimoto et al. [55]	37 (28/9)	Hemodialysis	Simvastatin (5 mg daily or 10 daily)	Control	16 weeks
Joy et al. [54]	45 (19/26)	Hemodialysis	Atorvastatin (10 mg daily, titrated to goal LDL-C reduction)	Placebo	36 weeks
Lullo et al. [43]	130 (80/50)	Creatinine clearance between 45 and 55 mL/min	Fluvastatin (80 mg daily)	Control	6 months
Manzano et al. [42]	76 (41/35)	Peritoneal dialysis	Pravastatin (20 mg daily)	Placebo	2 months
Mou et al. [57]	48 (25/23)	CKD	Pravastatin (20 mg daily)	Control	96 weeks
Panichi et al. [58]	55 (28/27)	CKD (3-4)	Simvastatin (40 mg daily)	Placebo	6 months
Ridker et al. [59]	3267 (1638/1629)	eGFR < 60 mL/min/1.73 m^2^	Rosuvastatin (20 mg daily)	Placebo	48 months
Sawara et al. [60]	48 (22/16)	eGFR < 90 mL/min/1.73 m^2^ and ≥15 mL/min/1.73 m^2^	Rosuvastatin (20 mg daily)	Control	12 months
Tugrul sezer et al. [61]	45 (25/20)	Peritoneal dialysis	Simvastatin (20 mg daily)	Placebo	1 month
Verma et al. [62]	83 (44/39)	eGFR < 60 mL/min/1.73 m^2^	Rosuvastatin (10 mg daily)	Control	20 weeks
Vernaglione et al. [63]	34 (16/17)	Hemodialysis	Atorvastatin (10 mg daily)	Placebo	6 months
Zagajewska et al. [56]	36 (18/18)	CKD (3-4)	Atorvastatin (20 mg daily)	Placebo	6 months

CKD: chronic kidney disease.
